# Where and How Extensive Are the Living Areas of the Elderly? An Empirical Study on ‘Ageing in Place’ in Three Small and Medium-Sized Japanese Cities

**DOI:** 10.3390/ijerph18115960

**Published:** 2021-06-02

**Authors:** Tatsuya Nishino

**Affiliations:** Faculty of Giosciences and Civil Engineering, Institute of Science and Engineering, Kanazawa University, Kanazawa 920-1192, Japan; tan378@se.kanazawa-u.ac.jp; Tel.: +81-76-234-4649

**Keywords:** ageing in place, the elderly, seniors, living area, small and medium-sized city, Japan

## Abstract

The policies regarding the elderly in advanced countries are based on the notion of ‘ageing in place’. The question arises, where and how extensive can the ‘place’ be? Is there a method of estimating a senior’s living area? The purpose of this study was to determine the common characteristics of the living areas of seniors in three small and medium-sized Japanese cities. The basic methodology involved a comparative analysis involving these cities. We used case studies to cross tabulate interviews regarding the daily outings of participants, some of whom needed long-term care while others did not. The data covered a total of 727 participants, 307 of whom needed long-term care and 420 requiring none. Comparative analysis revealed the common characteristics of living areas for seniors in these cities, i.e., two-layered living areas of healthy seniors; fewer outings on foot due to frailty; the average moving time via transportation is approximately 12 min; and living areas overlap districts where hospitals and stores are located. The results indicate that we can roughly estimate the living areas of seniors in any neighborhood to investigate accessibility to nearby hospitals and stores.

## 1. Introduction

One of the significant societal achievements has been the increase in longevity worldwide [1]. However, an increasingly ageing population presents a new challenge to countries in terms of meeting the needs of seniors and creating policy [2]. There is no doubt that elderly-care systems are important in an ageing society. Denmark, for example, is known for its highly developed social care system for the elderly; public care was introduced as an alternative to informal care by family members in the 1970s [3]. Since then, they have developed new attitudes towards old age and new ways of providing services to the elderly [4]. Their policies have encouraged seniors to stay in their own homes for as long as possible. In the Netherlands, on the other hand, a de-linking system of housing and care was developed to make seniors more autonomous and care providers more customer-oriented [5].

By 2050, most Asian countries will become ‘aged societies’, and this is known as ‘ageing Asia’ [6]. While the elderly in these countries have traditionally been cared for by their family members [7,8], previous studies have pointed out the need to change from traditional family support to public care systems [9,10,11]. Hiroi introduced three phases in social-security-system development with the notion of health transition [12], and Ohizumi categorized Asian countries on the basis of their social security systems and challenges, economic development, and social change [13].

In the first phase of health transition, the main problem is infectious diseases. The key issue is public health policy. Industrializing countries, such as India, Vietnam, Laos, Cambodia, and Myanmar, are categorized in this phase.

In the second phase, the disease structure changes to personal chronic diseases. Insurance systems are mainly used to finance social security, as well as medical services and hospitalizations. China, Malaysia, Thailand, Philippines, and Indonesia are categorized in this phase, which have been undergoing rapid industrialization.

The third phase is in the need for care services. The elderly need both medical and long-term care services. The two main shifts are from medical services to long-term care services and from institutional service to in-home service. Integration of the two shifts is the main topic of system design. Japan, South Korea, Taiwan, and Singapore are categorized in this phase. They have highly developed economies, low birth rates, and ageing populations.

As a country in the third phase, Japan has been developing its social welfare system since 1989 [14]. In 2000, the Japanese social care vision turned towards enabling the elderly requiring long-term care receive it in their homes by introducing the Long-Term Care Insurance Act. These polices are based on the notion of ‘ageing in place’ [15]. Seniors usually prefer to live in their familiar residences where they have memories [15]. In 2013, ‘the Community-based Integrated Care System’ was introduced with the revision of the Long-Term Care Insurance Act [16] to encourage people to continue to live in their hometowns to the end of their lives even if they require long-term medical care. It provides housing, medical services, long-term care services, prevention services, and livelihood support in an integrated manner in each community [17].

One of the main issues is how to define long-term care service areas for seniors. For the Community-based Integrated Care System, a service area is set by every municipality, the most familiar area based on comprehensive consideration of geographical conditions, population, traffic conditions, other social conditions, and the status of facility development to provide services subject to long-term care benefits, etc. According to the Ministry of Health, Labor and Welfare, the area should be centered around a junior high school district, which is considered to be 30 min or less by transportation. This guideline was introduced in the revision of the Long-Term Care Insurance Act in 2005 [18].

For implementation of ‘ageing in place’, we may ask why a junior high school district is considered a suitable living area for seniors. Although it might be difficult to establish care facilities in every neighborhood where the elderly live and are accustomed to, is it a suitable ‘place’ for the elderly to receive care services? When we say ‘ageing in place’, where and how extensive can the ‘place’ be? The answer might be different in each country, region, city, or neighborhood. Therefore, is there a method that can be applied to any area to estimate a senior’s living area? 

Several studies have been conducted on the living areas of seniors in Japan. Muronaga and Morozumi examined regional differences in the outing behavior of seniors living in Kumamoto city on the basis of a personal trip survey [19]. They also clarified that the living areas of seniors was expanded by using cars [20]. They also reported the difference in the elderly going out depending on the areal environment [21]. Takizawa and Yamamoto et al. clarified that the outing distances of relatively healthy seniors in the old, new, and rural areas in Oyama city, Tochigi Pref., differ [22,23,24].

The purpose of this study was to determine the common characteristics of the living areas of seniors from case studies in three small and medium-sized cities in Japan [25,26,27]. Our goal was to determine a general method of estimating senior living areas to contribute to health-care service provision for implementation of ‘ageing in place’. Our study also generalized the outings of seniors, including those requiring long-term care or support, making it possible to infer areal convenience due to differences in accessibility to essential facilities.

## 2. Materials and Methods

The definition of ‘long-term care required’ is a condition that requires continuous physical care, and long-term care services are available. There are 5 levels of those who require long-term care depending on severity. ‘Support required’ is expected to interfere with daily activities but not with the need of physical care, and preventive services are available to improve or maintain the current condition. There are two levels for ‘support required’ depending on severity [28]. 

The certification for long-term care has two steps [29]. The first step is a computer-aided automatic judgment on the basis of a physical and mental condition survey conducted by a certified investigator of the municipality and the opinion of the attending medical doctor. The second step is an examination by a nursing care certification examination committee composed of academic experts in health, medicine, and welfare.

**Figure 1:**
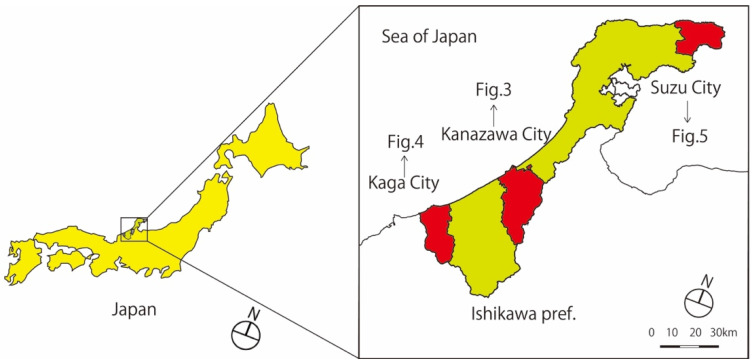
Target cities for this study.

The basic methodology involved a comparative analysis of case studies in three small and medium-sized cities in Ishikawa Prefecture, Japan—Kanazawa, Kaga, and Suzu [25,26,27] (Figure 1). 

### 2.1. Outlines of Three Target Cities

**Figure 2:**
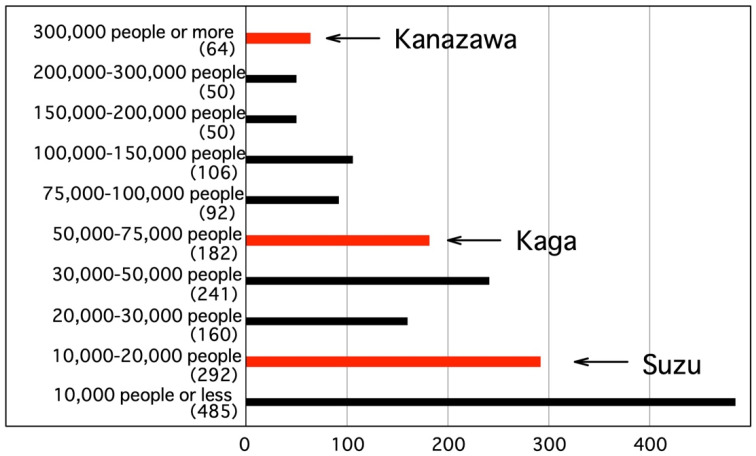
Populations of target cities and nationwide ranking (unit: persons).

The three target cities were chosen in consideration of differences in population and location. Table 1 shows the population, area, population density, and ranking among all 1741 municipalities in Japan of the three cities. Figure 2 shows the population ranking of the three cities out of all municipalities in Japan. The population ranking varies with Kanazawa in the top 2.4%, Kaga in the top 24.0%, and Suzu in the top 62.7%. The areas are rather large, all three cities in the top 13.1 to 28.4%. The population density varies with Kanazawa in the top 21.4%, Kaga in the top 47.5%, and Suzu in the top 74.0%.

**Figure 3:**
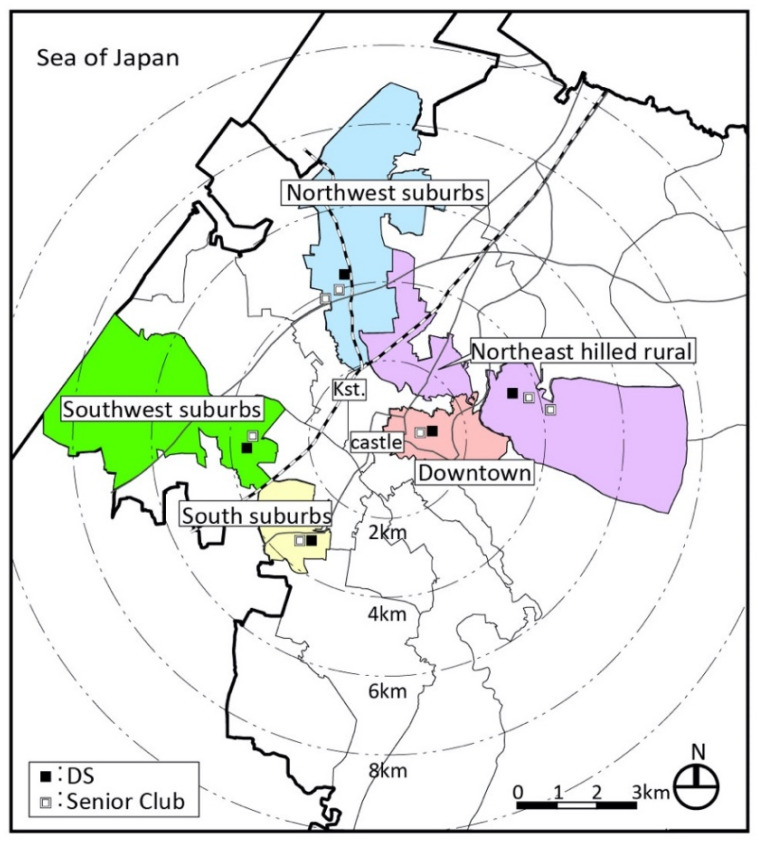
Five target districts in Kanazawa (unit: kilometers).

Kanazawa has an urban structure centered on the old castle ruins and has diverse regional characteristics (Figure 3). There are 21 secondary medical institutions (hospitals) and 2 tertiary medical institutions (the university hospital and prefectural hospital). There are two department stores and a large shopping center (SC) in the center and many stores along suburban main roads. There are many supermarkets in each district.

**Figure 4:**
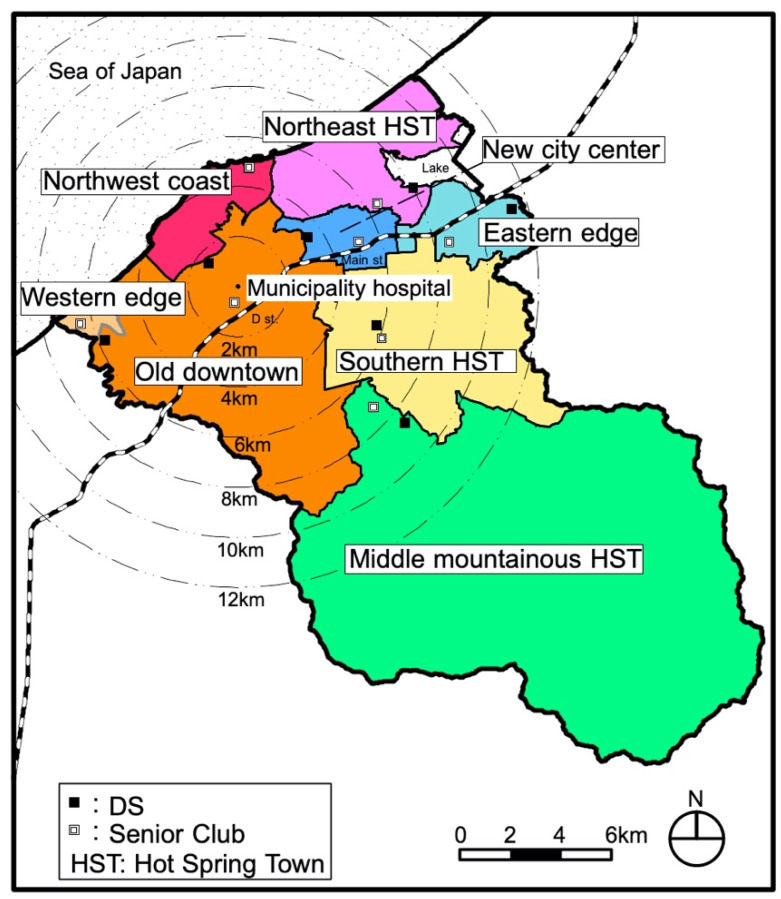
Eight target districts in Kaga (unit: kilometers).

Kaga has an urban structure with multiple urban cores of old towns (Figure 4). There are two secondary medical institutions (hospitals) in the center of the old city and one in the former Yamanaka town (middle mountainous hot spring town district). There is a large SC in the center of the new city, one in the old downtown nearby, and a total of 17 supermarkets are located in the urban core of each district except the northwest coast district, western district, and the new city center district.

**Figure 5:**
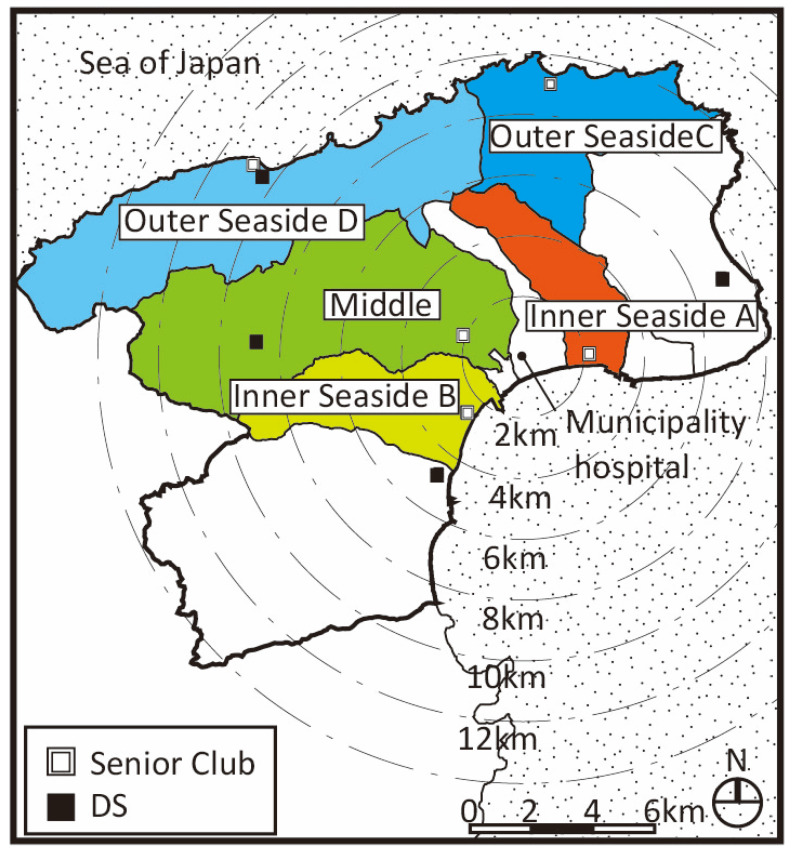
Five target districts in Suzu (unit: kilometers).

Suzu is located at the outer tip of Noto Peninsula, the north side (Sea of Japan side) of the city is called “Outer Seaside” and the south side is called “Inner Seaside”. It is a mono core urban structure where a city hall and a municipal hospital are in Inner Seaside (Figure 5). There is only one secondary medical institution as the municipal hospital, one large SC, and five supermarkets or drugstores all in Inner Seaside.

Table 2 lists the number of care facilities in the three cities.

### 2.2. Survey Method

We selected five districts in Kanazawa, eight districts in Kaga, and five districts in Suzu in consideration of the distribution balance in each city; downtown, suburb, and periphery areas. In Kaga, we conducted research in all districts upon request of the municipality. In each district, a day-care service center (DS) and a community group or a seniors’ club dedicated to preventing the need for care were selected on the basis of the municipality office’s recommendation. Since we wanted to collect as much data as possible, we recruited around 15 DS users (long-term care or support required) on the basis of recommendations from DS managers and at least 20 community group or seniors’ club users (for healthy seniors). We then conducted interviews and questionnaires on the attributes, home address, and places visited daily (supermarkets, clinics, hospitals, beauty salons or barber shops, banks, restaurants, DSs, etc.) of the participants living in each district. A questionnaire survey for the managers in the DSs was also conducted regarding the place of residence and attributes (age, sex, living in single or with family, degree of care required) of registered users.

We collected the data of 175 seniors in Kanazawa (71 needing long-term care or support and 104 who did not), 323 seniors in Kaga (149 requiring long-term care or support and 174 who did not), and 229 seniors in Suzu (87 requiring long-term care or support and 142 who did not). The total was 727 seniors, 307 of whom required long-term care or support and 420 not requiring any in the three cities. (Table 3). The interviews were conducted in Kanazawa from September 2011 to November 2012, in Kaga from September to October 2014, and in Suzu from August to September 2015. Although the Community-based Integrated Care System was introduced in 2013, there was no change for current DS service users and their living arrangement.

### 2.3. Analysis Method

The living area of each participant was identified by plotting the place of residence and places visited on a geographic information system (GIS) map, which were obtained from the above interviews. From those map data, the distance between each participant’s home and each place visited was calculated as a spatial distance. Since straight-line distance was often used in previous studies, we also adopted it for our study and calculated outing distances. Statistical management status was taken into consideration so that extremely distant outings by specific individuals would not push up the average outing distance of the entire district. We also calculated the single trip time-distances between each participant’s home and each outing location. On the basis of the above data, we analyzed the characteristics of the living area of the participants by district, comparing those requiring support or care with those requiring none, and by means of transportation. We also clarified the characteristics of going out for those requiring support or care. By comparing the results of the interviews among the participants in the three cities, the common characteristics regarding the living areas of seniors were determined.

### 2.4. Characteristics of Participants

Table 3 lists the characteristics of the participants. Of the 727 participants, the female to male ratio was 3.9, much higher than the nationwide ratio for those age 65 or older, i.e.,1.31 [30]. It was much higher than the target cities’ ratios, 1.36 in Kanazawa, 1.43 in Kaga, and 1.48 in Suzu [31]. Our data had a large gender bias to female.

The average age was 85.7 for those who needed support or care and 78.7 for those who did not. This corresponds with the evidence that almost 90% of those receiving support or care are over 75 years old in Japan [32].

In our study, 42.2% of the participants needed support or care, and 57.8% did not. The percentage of participants requiring support or care was high compared with the nationwide percentage certified as requiring support or care aged 65 and over, i.e., 18.0% in October 2015 (6036 people/33,464 people) [33].

Therefore, if a simple arithmetic mean is taken when averaging in each district, the results of those requiring support or care will be overly reflected, so we calculated the weighted average on the basis of the actual proportion of those requiring support or care and those who did not in each district.

The average level of care required for those requiring care was 1.5 out of 5 (level 5 is the most in need). The overall household composition was 19.8% living alone, 23.5% couples, and 56.2% living with families. Comparing this with the 2014 national data, living alone is almost the same, there are fewer elderly couples, and there are more families living together (The nationwide percentage of elderly households was 17.4% living alone, 38.0% for couples, 40.6% living with children, and 3.9% living with relatives.) [34].

## 3. Results

We compared the daily living areas of the participants living in the three cities (Kanazawa, Kaga, Suzu) and determined common characteristics.

The correlation between the average outing distance and level of care required in district was only observed in Kaga, thus, not always a correlation (Table 4). This means that outing distance has no correlation with physical condition. 

There is a strong correlation between average numbers of places visited on foot and female to male ratios for both those requiring care (R^2^ = 0.82) and those requiring none (R^2^ = 0.77). There is also a strong correlation between average numbers of places visited by transportation and female to male ratios for both those requiring care (R^2^ = 0.84) and those requiring none (R^2^ = 0.99).

### 3.1. Participants Requiring Care Stay within 500 m When Going Out on Foot

**Figure 6:**
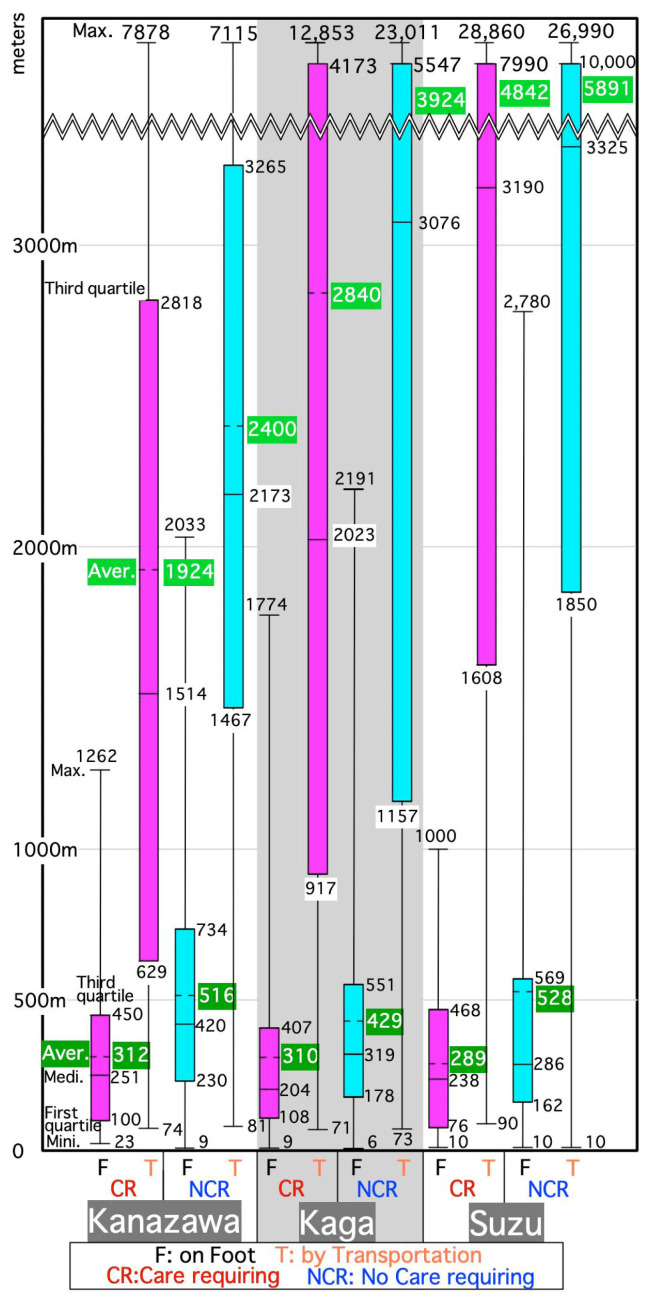
Distribution of all single outing straight-line distances of participants in three target cities (unit: meters).

Figure 6 is a box-and-whisker graph showing the total outing distance for each participant requiring care or not requiring care in the three cities. From the first to third quartiles in Figure 6 going out on foot was within 500 m for those requiring care. The distance for participants not requiring care was slightly longer. This might be because bicycles were also included in the walking category as self-movement.

### 3.2. Two-Layered Living Area of Seniors

While the box from the first to third quartiles of outing distance on foot is in 76–734 m, boxes of outing distance by means other than walking extends to 629–10,000 m, as shown in Figure 6. Therefore, the two layers of the walking area around the home and the wider area using transportation seemed to be common to all participants. This was found in about 60% of non-care requiring seniors and those requiring care in Kanazawa [25], about 80% of non-care requiring seniors in Kaga [26], and 70% of those in Suzu [27].

For those requiring care, however, a two-layered living area was found mainly in downtown Kanazawa, but not in Kaga or Suzu (Table 4). This is considered due to the fewer outings on foot in the next section.

### 3.3. Fewer Outings on Foot by Those Requiring Care

The average number of places visited on foot by participants who required care in Table 4, are about 20–30% the number of places visited by those who did not require care. That is, there were few places to go out within walking distance for people requiring care. Therefore, fewer outings on foot by those requiring care were observed in all three cities. It is almost impossible for people requiring care to find a two-layered living area because they do not go out on foot much. Instead, there was a strong dependence on transportation even within walking distance in Kaga and Suzu.

There were also areas where we could observe fewer outings on foot than other areas due to geography. For example, there are many slopes in some areas (e.g., Northeast hilled rural district of Kanazawa) [25], and there are no places to go in such areas (the Middle, Outer Seaside districts C and D in Suzu) [27], so even non-care requiring seniors went out less on foot. In these districts, the number of places to go on foot for seniors requiring care is also small (0.6 in the Northeast hilled rural district of Kanazawa [25], and in Suzu, 0.4 in the Middle district, 0.3 in Outer Seaside C, and 0.9 in Outer Seaside D [27]. It is mainly in downtown where a two-layered living area can be observed by those who require care in Kanazawa. The number of places to go on foot for these seniors in this district is the highest in all districts with an average of 2.2. This might indicate the convenience of this district [25].

### 3.4. Average Single Outing Straight-Line Distances

**Figure 7:**
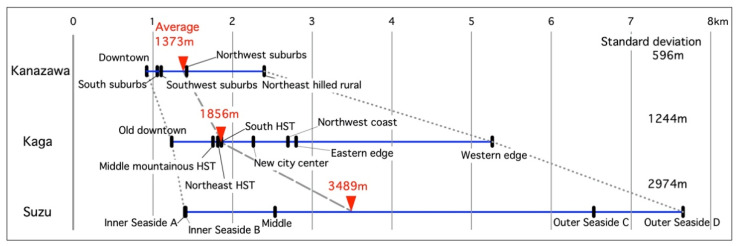
Distribution of proportion-weighted average of SOSLDs in each district of three target cities (unit: kilometers).

We compared the average single outing straight-line distances (SOSLDs) by district among the three cities. Figure 7 shows the distribution of the proportion-weighted averages (by proportion of population of seniors requiring support or care and those not requiring it) (PWAs) of the SOSLDs in each district of the three cities. The weighted average PWA of the SOSLDs by district population in each city are 1373 m in Kanazawa, 1856 m in Kaga, and 3489 m in Suzu. This order is inversely proportional to population and density of these cities. However, the minimum PWAs of the SOSLDs in each city are not as different. These districts are in the administrative center of each city. The maximum PWAs of the SOSLDs in each city, however, increased in the same order as the weighted average PWA of the SOSLDs by district population in each city.

The standard deviations indicate that the variation increases in the same order as the weighted average PWA of the SOSLDs by district population of each city. In other words, this increase is inverse to the population and population density, but this might be due to the difference among districts with the smallest and largest PWAs of the SOSLDs in each city. The difference between the smallest and largest districts is small in Kanazawa but large in Kaga and Suzu.

Regarding the maximum PWAs of SOSLDs, the western edge of Kaga forms the southwestern edge of Ishikawa Pref., and Outer Seaside C and D in Suzu form the northeastern edge of the prefecture, so the living area widens at the edge of the prefecture. Excluding these prefectural edge districts and central districts, the PWAs of the SOSLDs for each district are within 1–3 km; within 1.5 km in Kanazawa but around 2.5 km in Kaga and Suzu. In other words, compared with Kanazawa, Kaga and Suzu have wider living areas.

**Figure 8:**
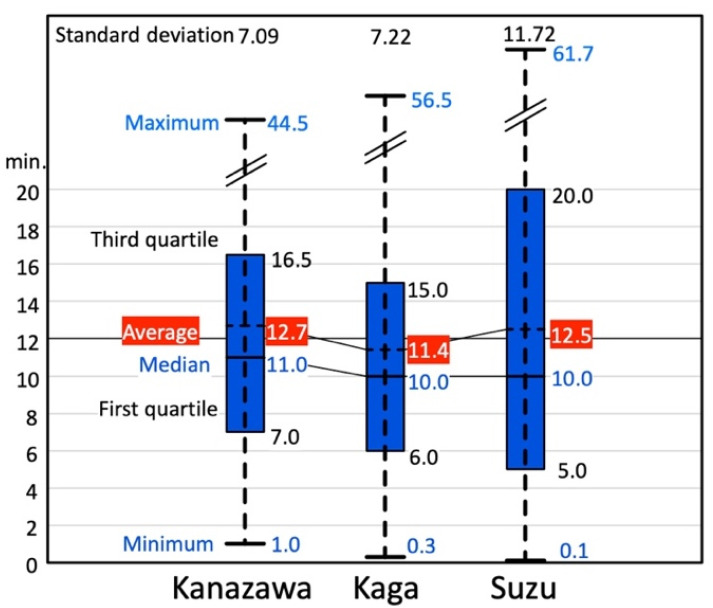
Distribution of all single outing times for participants in three target cities by transportation (unit: minutes).

Figure 8 shows the distribution of the single outing time-distances for participants in the three cities by transportation. The average is around 12 min for all cities.

### 3.5. Essential Facilities That Increase Average Outing Distance

We compared the facilities that increased the average outing distances of the participants. These facilities might be essential for seniors because they visit them even when they are far from their homes. Table 5 shows a comparison among the three cities regarding the top five facilities in order of greater impact on extending the outing distances of seniors requiring support and those not requiring it. These are the sums of the outing distances (equal to average by frequency) for each outing destination in descending order.

For seniors not requiring support or care, the top facilities were all stores. The second and lower rankings differed in each city: restaurant, place for leisure activities, hospital, bank, and salon. 

The top five facilities for those requiring support or care were common in the three cities, i.e., clinic, hospital, DS, store, and salon. These destinations might be essential for seniors requiring support or care because the average number of outings for these seniors is about 4–5, as shown in Table 4.

### 3.6. Overlap of Living Areas

**Figure 9:**
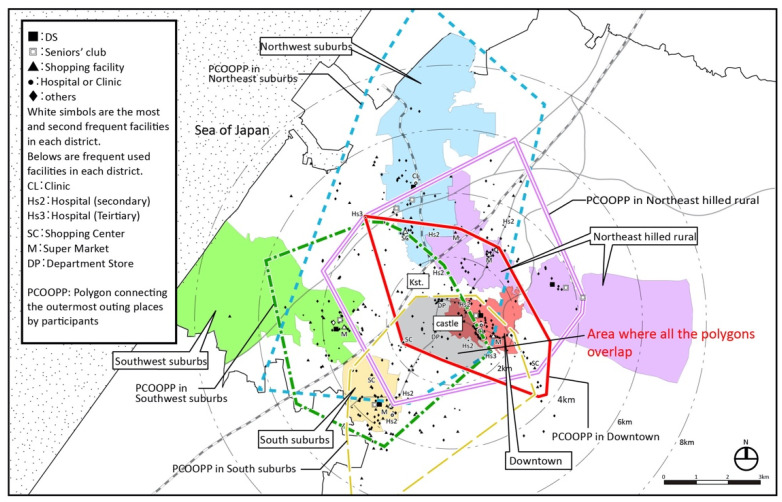
Polygons connecting outermost places visited by participants in each district in Kanazawa.

We now look at the overlapping of living areas. If one draws a polygon that connects the outermost places visited by participants in each district, every polygon in Kanazawa is biased from each district to the city center (geographically central), thus showing a “center eccentricity” of the living areas (Figure 9) [25]. The reason might be that the major hospitals, stores, and two department stores are located in the center, which were outings from all five districts. 

**Figure 10:**
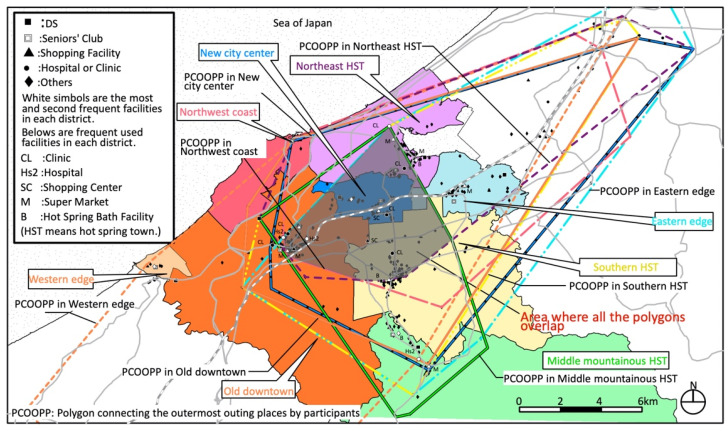
Polygons connecting outermost places visited by participants in each district in Kaga.

The area where the outing areas overlap in Kaga emerged as a combination of urban cores, such as the old downtown, new city center, northeast hot spring town, and southern hot spring town district (Figure 10) [26]. This is called “distributed concentration in multiple urban cores” in the living area. In particular, seniors not requiring support or care frequently went to stores (including large SCs) in the old downtown and new city center districts, and those requiring support needed the most use of hospitals in the old downtown. 

**Figure 11:**
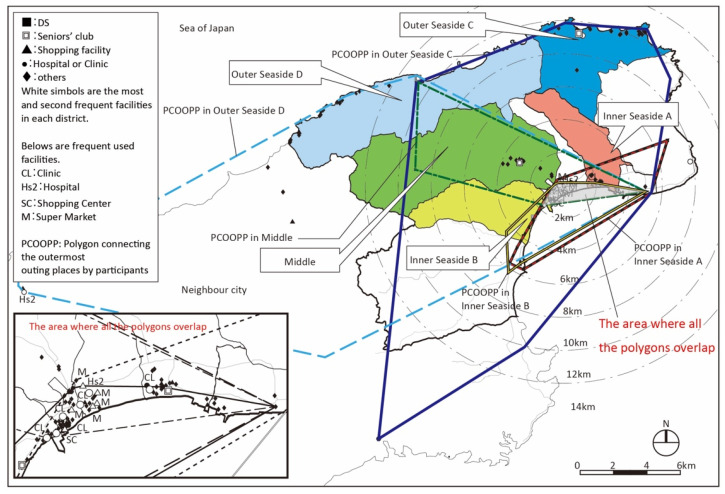
Polygons connecting outermost places visited by participants in each district in Suzu.

In Suzu, all the outing areas included the area around the city center in Inner Seaside (Figure 11) [27]. This characteristic is defined as the “unipolar concentration” of the living area. The hospital and stores are located in this area.

## 4. Discussion

Regarding going out on foot in Section 3.1., the area for a housewife to go and do daily shopping is generally 400–500 m [35] and the distance that the elderly can walk comfortably is 390 m (0.67 m/s in 9.7 min) [36]. Therefore, it might be common for most seniors requiring care to stay within 500 m of their residence when going out on foot.

With reference to two-layered living area of seniors in Section 3.2., Muronaga and Morozumi clarified that the living area of seniors expanded due to using cars in Kumamoto city [20], which is a larger regional city with a population of 740204 people, area of 390.32 km^2^, and population density of 1889 persons per km^2^ in 2014 [37]). In our study, we found a two-layered living area for healthy seniors. There were also cases in which there is no such area because of geography, even for healthy seniors. Seniors requiring support usually go out to few places on foot except in convenient districts.

As for average single outing straight-line distances in Section 3.4., in a survey of healthy seniors in Oyama city (population: 165,484 people, area: 171.61km^2^, population density: 964.3 persons per km^2^ in 2015 [38]), Tochigi Pref., the distribution of the average SOSLDs was found in the old downtown; 0.7–0.8 km, new downtown; 1.0–1.1 km, and rural area; 3.3–4.1 km [22,23,24]. The old and new downtowns of Oyama are almost the same as the central districts in this study, and the rural area of Oyama is between the edge and other districts of our study. Therefore, we can extract hypothesize that the average SOSLD is around 1 km in downtown areas and increases as into rural areas.

It is interesting that although the distributions of the average SOSLDs in the three cities are dispersed, the distribution of average single outing time-distance is around 12 min. This might be because the time-distance calculation takes into account traffic jams and signals in urban areas, so if one drives in a rural area, one can go much farther than in an urban area in the same amount of time. The average daily living time-distance is around 40 min for all generations in Japan [39]. Therefore, our result for seniors is much shorter than that of the all-generation average. The general application of the average time may be common in urban areas in small and medium-sized Japanese cities because it depends on geography.

We examine essential facilities that increase average outing distance in Section 3.5. Each participant went to at least a DS because we selected support or care requiring participants who were registered at DSs. The outings that had a greater impact on extending the outing distance than DSs were hospitals, stores, and clinics in Kanazawa, and only the hospital in Kaga (Table 5). In Suzu, the DS was the highest, and the hospital was second. In other words, the impact of the hospital is lower than that of the DS in Suzu compared with that in Kaga. 

The reason is thought to be that the DS in Suzu is quite far away. The average distance to a DS is 5099 m but 1967 m in Kaga and 929 m in Kanazawa. Since the DS is a place where all participants went, if the distance between a DS and their home is long, it will have a significant impact on increasing the average outing distances of seniors requiring support. 

It is hypothetically possible that the participants in Suzu do not use the hospital as much as those in Kaga. Since the ratio of the total frequency of going to a hospital to seniors requiring support or care in Suzu is higher than that in Kaga and the same as that in Kanazawa (Table 5), the above hypothesis is rejected.

The average distance to a beauty salon is 971 m in Kanazawa, 1190 m in Kaga, and 1342 m in Suzu, which shows that these seniors use nearby salons. The ratio of the total frequency of going to SCs to seniors requiring support or care (Table 5) in Suzu is considerably lower than that of Kanazawa and Kaga. In Kaga, some seniors living with families said, “I leave shopping to my son’s wife living with me”. About half of single-person households were thought to ask their children who live separately or home helpers for shopping. In Kaga, seniors requiring support or care depended on others for shopping, and the same tendency can be seen in Suzu.

To summarize the above, for seniors not requiring support or care, the SC had the greatest impact on increasing the average outing distance, but for seniors requiring it, the clinic, hospital, and DS had the greatest impact. This tendency might be general because stores are essential for all seniors. Regarding seniors requiring support or care, the clinic, hospital, and DS might be common in countries where there is easy accessibility to medical and care services. We can roughly estimate the convenience of living areas for seniors from accessibility of these facilities.

And from Section 3.6., the area where stores (including large SCs) and hospitals are located is determined as where the living areas of seniors overlap. It might be common in small cities where the living areas of seniors overlap around hospitals because the number of hospitals is limited in small cities.

## 5. Conclusions

Our goal was to determine a general method of estimating living areas of seniors for the implementation of ‘ageing in place’. Therefore, we determined the following common characteristics of such living areas by comparing the interview results from the three small and medium-sized Japanese cities of Kanazawa, Kaga, and Suzu and their possibility for general application:

- Most seniors requiring support or care walk out within 500 m;
- Two-layered living area with the walking area around the home and the wider area using transportation were observed for over half of healthy seniors;
- There are fewer outings on foot due to frailty;
- The average outing time-distance by transportation is approximately 12 min;
- The destinations extending the outing distance the most for people requiring support or care are hospitals and care facilities and shopping facilities for people not requiring support or care;
- The living areas of seniors overlap where hospitals and shopping facilities are located.

This study has some limitations. The results are from case studies in small and medium sized cities. This means that some results, especially distances using transportations, might be different in metropolitan areas where public transportation is commonly used. This is one of our further research topics. Detail examination on the effects due to gender bias is also another further research issue.

Some of our findings depend on the Japanese societal context. For example, we have almost ‘free access’ to hospitals. We normally need an introduction document from our general practitioner, but if we pay an extra fee, this document is not needed. Medical accessibility differs in each country; therefore, it is possible to interpret ‘hospital’ in this research as ‘general practitioner’s clinic’ in societies where medical accessibility is strictly controlled.

However, medical services and food shopping are essential living activities that may be common to all seniors. Therefore, accessibility to these service facilities extend senior’s living areas.

To understand what determines living areas of seniors is fundamental for the implementation of ‘ageing in place’. It is most important to be able to roughly estimate the living areas of seniors in any neighborhood to look at accessibility to hospitals and shopping facilities. This can be applied to policy making not only for setting care-service areas but also for location planning for senior-care facilities, hospitals, and senior homes, as well as planning public transportation routes.

We can imagine a future when it will be even more common to receive medical care and shop for food online. However, this will take time.

## Figures and Tables

**Table 1:** Population, area, and population density of three target cities.

City	Kanazawa	Kaga	Suzu
Population (persons) (October 2015)	465,810	67,235	14,631
Ranking	428 (2.4%)	417 (24.0%)	1091 (62.7%)
Area (km^2^) (October 2016)	468.64	305.54	247.2
Ranking	228 (13.1%)	389 (22.3%)	494 (28.4%)
Population Density (persons per km^2^)	993.96	219.82	59.19
Ranking	372 (21.4%)	827 (47.5%)	1289 (74.0%)

**Table 2:** Numbers of senior care-service areas, junior high school districts, and care facilities in three cities.

City	Kanazawa	Kaga	Suzu
Number of senior care service areas	24	7	1
Number of junior high school districts	19	6	4
Number of nursing homes	19	10	2
(capacity)	(1852 beds)	(440 beds)	(100 beds)
Number of dementia group living homes	49	12	4 (7units)
(capacity)	(932 beds)	(177 beds)	(63 beds)
Number of community-based multi-function facilities	24	12	1

**Table 3:** Characteristics of participants in three target cities.

City		Participants Percentage for All (%) (Persons)	Female/Male (Male; Female)	Living in Single; Couple; with Family; Senior House When Singles as 1.0 (Raw Numbers)	Average Age (Years Old)	Average Degree of Care-Requiring
Kanazawa	Care-requiring senior	9.8% (71)	3.2 (17; 54)	1; 0.5; 1.9; 0 (21; 10; 40; 0)	84.8	1.4
	No care-requiring senior	14.3% (104)	7.0 (13; 91)	1; 1.1; 1.9; 0 (26; 28; 50; 0)	78.4	-
	Total	24.1% (175)	4.8 (30; 145)	1; 0.8; 1.9; 0 (47; 38; 90; 0)	-	-
Kaga	Care-requiring senior	20.5% (149)	2.9 (38; 111)	1; 0.5; 3.2; 0.1 (31; 17; 98;3)	85.2	1.5
	No care-requiring senior	23.9% (174)	8.7 (18; 156)	1; 1.2; 2.6; 0 (36; 43; 95; 0)	78.3	-
	Total	44.4% (323)	4.8 (56; 267)	1; 0.9; 2.9; 0 (67; 60; 193; 3)	-	-
Suzu	Care-requiring senior	12.0% (87)	2.6 (24; 63)	1; 1.1; 3.7; 0 (15; 17; 55; 0)	87	1.7
	No care-requiring senior	19.5% (142)	2.7 (38; 104)	1; 0.7; 1.7; 0 (42; 29; 71; 0)	79.3	-
	Total	31.5% (229)	2.7 (62; 167)	1; 0.8; 2.2; 0 (57; 46; 126; 0)	-	-
The three cities	Care-requiring senior	42.2% (307)	2.9 (79; 228)	1; 0.7; 2.9; 0 (67; 44; 193; 3)	85.7	1.5
	No care-requiring senior	57.8% (420)	5.1 (69; 351)	1; 1; 2.1; 0 (104; 100; 216; 0)	78.7	-
	Grand total	100% (727)	3.9 (148; 579)	1; 0.8; 2.4; 0 (171; 144; 409; 3)	-	-

**Table 4:** Factor comparison of three target cities.

City		Kanazawa	Kaga	Suzu
Relation between outgoing distance and level of care requiring (R^2^)		0.40	0.83	0.32
Two- layers of living area	No care-requiring senior	observed	observed	observed
	Care-requiring senior	Downtown only	no	no
Fewer outgoings on foot	Care-requiring senior	observed	observed	observed
Dependence on transportation	Care-requiring senior	observed	strong	strong
Average number of places visited on foot	No care-requiring senior	4.5	4.1	2.8
	Care-requiring senior	1.3	0.7	0.6
Average number of places visited by transportation	No care-requiring senior	8.9	9.5	7.9
	Care-requiring senior	5	4	3.8

**Table 5:** Five major factors that extended outing distances of participants in three cities.

City		Kanazawa	Kaga	Suzu
Top five facilities for outgoing of non care-requiring senior	1	Shopping store	Shopping store	Shopping store
	2	Restaurant	Restaurant	Secondary Hospital
	3	Place for leisure activities	Place for leisure activities	Clinic
	4	Secondary or Tertiary Hos.	Clinic	Bank
	5	Bank	Secondary Hospital	Beauty salon
Top five facilities for outgoing of care-requiring senior	1	Secondary or Tertiary Hos.	Secondary Hospital	DS
	2	Shopping store	DS	Secondary Hospital
	3	Clinic	Clinic	Clinic
	4	DS	Shopping store	Shopping store
	5	Beauty salon	Beauty salon	Beauty salon
Ratio of the total frequency of going to a hospital to seniors requiring support or care		0.61 (0.55) *	0.50 (0.49) *	0.61 (0.61) *
		* shows the rate of outpatients/participants		
Ratio of the total frequency of going to SCs to seniors requiring support or care		0.81 (0.46) *	0.57 (0.37) *	0.34 (0.24) *
		* shows the rate of users/participants		

## Data Availability

The data supporting this meta-analysis were extracted from previously published studies, which have been cited in this paper.
